# 6-Arylimino-2-(2-(1-phenylethyl)naphthalen-1-yl)-iminopyridylmetal (Fe and Co) Complexes as Highly Active Precatalysts for Ethylene Polymerization: Influence of Metal and/or Substituents on the Active, Thermostable Performance of Their Complexes and Resultant Polyethylenes

**DOI:** 10.3390/molecules25184244

**Published:** 2020-09-16

**Authors:** Wenhua Lin, Liping Zhang, Jiahao Gao, Qiuyue Zhang, Yanping Ma, Hua Liu, Wen-Hua Sun

**Affiliations:** 1 School of Textiles Science and Engineering, Jiangnan University, Wuxi 214122, China; 6180709011@stu.jiangnan.edu.cn; 2 Key Laboratory of Engineering Plastics and Beijing National Laboratory for Molecular Science, Institute of Chemistry, Chinese Academy of Sciences, Beijing 100190, China; gaojh514@163.com (J.G.); zhangqiuyue@iccas.ac.cn (Q.Z); myanping@iccas.ac.cn (Y.M.); hualiu@iccas.ac.cn (H.L.); 3 State Key Laboratory for Oxo Synthesis and Selective Oxidation, Lanzhou Institute of Chemical Physics, Chinese Academy of Sciences, Lanzhou 730000, China

**Keywords:** linear polyethylenes, cobalt precatalyst, iron precatalyst, thermostable and efficient catalysis, correlation between structure and activity

## Abstract

A series of 6-arylimino-2-(2-(1-phenylethyl)naphthalen-1-yl)iminopyridines and their iron(II) and cobalt(II) complexes (**Fe1**–**Fe5**, **Co1**–**Co5**) were synthesized and routinely characterized as were **Co3** and **Co5** complexes, studied by single crystal X-ray crystallography, which individually displayed a distorted square pyramidal or trigonal bipyramid around a cobalt center. Upon treatment with either methyluminoxane (MAO) or modified methyluminoxane (MMAO), all complexes displayed high activities regarding ethylene polymerization even at an elevated temperature, enhancing the thermostability of the active species. In general, iron precatalysts showed higher activities than their cobalt analogs; for example, 10.9 × 10^6^ g(PE) mol^−1^ (Co) h^−1^ by **Co4** and 17.0 × 10^6^ g(PE) mol^−1^ (Fe) h^−1^ by **Fe4**. Bulkier substituents are favored for increasing the molecular weights of the resultant polyethylenes, such as 25.6 kg mol^−1^ obtained by **Co3** and 297 kg mol^−1^ obtained by **Fe3**. A narrow polydispersity of polyethylenes was observed by iron precatalysts activated by MMAO, indicating a single-site active species formed.

## 1. Introduction

Late-transition metal precatalysts have made great progress toward ethylene polymerization since the pioneering studies initiated by Brookhart and Gibson [1,2,3,4,5,6]. In addition to the α-diimino-nickel and palladium precatalysts [7,8,9,10,11,12], iron and cobalt precatalysts have showed a higher activity in ethylene polymerization and generated polyethylenes with highly linear structures [13,14,15,16,17,18]. However, the poor thermostability of iron and cobalt precatalysts and broad molecular weight distribution of obtained polyethylenes impeded further applications in the industry [19,20].

**Scheme 1:**
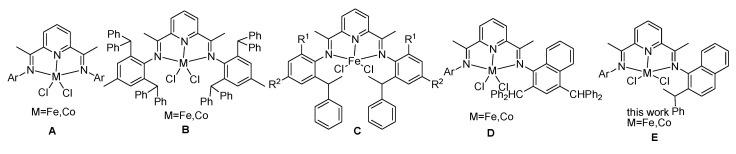
Structural variations in bis(imino)pyridine-iron and cobalt chloride precatalysts (**A**–**E**).

Though new frameworks have been developed for ligands supporting iron and cobalt species in ethylene polymerization, the model precatalysts (**A**, Scheme 1), namely the Gibson–Brookhart catalyst [1,2,3], are extensively investigated and improved catalytic performances through finely turning the steric and electronic influences of ligands used [21,22,23,24,25,26,27,28,29,30,31]. For example, benzhydryl-substituted precatalysts (**B**, Scheme 1) maintained high activities at the reaction temperature up to 80 °C [32,33], which fixed the critical problem regarding the thermostability of their analogs (**A**, Scheme 1) [1,2]. The successful precatalysts [32,33], in correcting the simulation results, provided evidence that the steric hindrance of ligands enhanced the thermal stability but decreased the catalytic activity of their complexes [34]. In our benzhydryl-modification [32,33], the phenylethyl-substituted precatalysts (**C**, Scheme 1) also improved the thermostability and maintained reasonable activities in ethylene polymerization [29]. Using naphthalenamine derivatives instead of anilines, the precatalysts (**D**, Scheme 1) also showed good catalytic performances regarding both thermostability and catalytic activity [13,26], showing positive influences in comparison to precatalyst **B** [32,33]. Subsequently, 2-(1-phenylethyl)naphthalenamine derivatives were recently developed for *N,N*-bidentate iron and cobalt complexes as active precatalysts for diene polymerization [35], and the substituent at the ortho-position of the *N*-aryl group heavily affected the catalytic performance. The 6-arylimino-2-(2-(1-phenylethyl) naphthalen-1-yl)imino pyridine derivatives and their iron and cobalt chlorides (**E**, Scheme 1) have been extensively synthesized and characterized. The title complexes show high activities in ethylene polymerization along with a good thermostability. The metal (iron or cobalt) and steric influences of ligands have been investigated and reported herein.

## 2. Results

### 2.1. Synthesis and Characterization

**Scheme 2:**
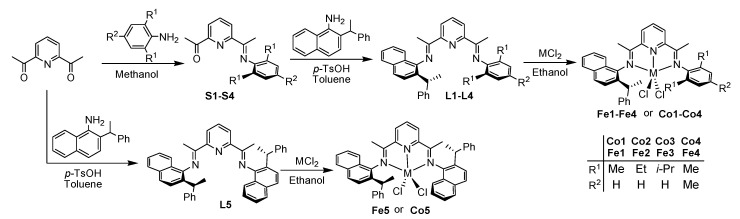
Synthesis route of the ligands (**L1**–**L5**) and their complexes **Fe1**–**Fe5** and **Co1**–**Co5**.

The 6-arylimino-2-acetylpyridine derivatives (**S1**–**S4**, Scheme 2) were prepared according to a previous procedure [36,37,38], and further reacted with 2-(1-phenylethyl)-1-naphthalenamine to form the bis(imino)pyridine ligands 6-arylimino-2-(2-(1-phenylethyl)naphthalen-1-yl)iminopyridine (**L1**–**L4**, Scheme 2) with a reasonable isolated yield (Scheme 2). The 2,6-bis((2-(1-phenylethyl) naphthalen-1-yl)-iminopyridine (**L5**) was obtained by reacting 2,6-diacetylpyridine with 2 equivalent of 2-(1-phenylethyl)-1-naphthalenamine. All iron (II) and cobalt (II) complexes were prepared by the stoichiometric reaction of the ligands (**L1**–**L5**, Scheme 2) with FeCl_2_·4H_2_O or CoCl_2_·6H_2_O, respectively, and further characterized by FT IR spectroscopy. Compared to free ligands (**L1**–**L5**), the stretching vibrations of C=N bonds in these iron and cobalt complexes have shifted to lower wavenumbers (1632–1640 cm^−1^ vs. 1615–1621 cm^−1^), which is consistent with the effective coordination between the metal (II) and imino nitrogen atoms [39]. The molecular structures of complexes **Co3** and **Co5** were further confirmed by single-crystal X-ray diffraction.

### 2.2. X-ray Crystallographic Studies

**Figure 1:**
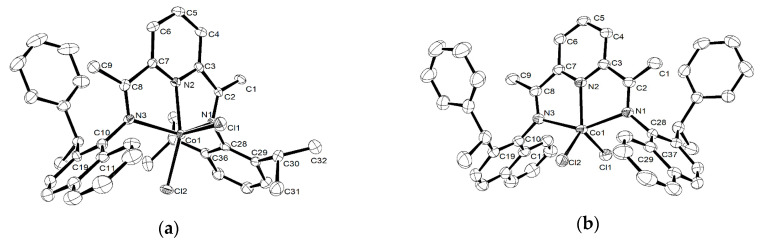
ORTEP drawing of **Co3** (**a**) and **Co5** (**b**) with thermal ellipsoids at the 30% probability level. Hydrogen atoms are omitted for clarity.

Single crystals of **Co3** and **Co5** (Figure 1) of suitable quality for the X-ray determinations were grown by the slow diffusion of diethyl ether into a solution of the corresponding complex in dichloromethane at room temperature. **Co3** and **Co5** comprise a single cobalt center surrounded by three nitrogen atoms of a tridentate ligand (N1, N2, and N3) and two chlorides (Cl1 and Cl2) to form a pentacoordinate geometry. Three nitrogen atoms and a Cl2 atom of **Co3** formed a basal plane, and the cobalt atom lay at a distance of 0.586 Å above this basal plane (Figure 1a). The dihedral angle of the naphthyl plane and the basal plane is 74.71°, and 80.75° for the angle between the benzene plane and the basal plane. **Co5** displayed C_2_ molecular symmetry (Figure 1b), and two naphthyl planes were close vertical to the pyridyl frame plane with dihedral angles are 78.50° and 85.39°, respectively. Two benzene substituents planes on naphthyl planes. Similar to many bis(imino)pyridine-cobalt complexes, the Co-N_pyridyl_ bond length of two complexes [**Co3**: 2.050(4) Å; **Co5**: 2.021(4) Å] is shorter than their exterior Co-N_imino_ ones [2.170(4)-2.294(4) Å], indicating good donor property of the central pyridine [13,14]. Other selected bond lengths and angles are listed in Table 1 and more detail can be seen in Supplementary Materials.

### 2.3. Ethylene Polymerization

Previous achievements have approved the efficiency of both MAO and MMAO cocatalysts in activating the iron or cobalt precatalysts for ethylene polymerization [40,41,42,43]. Therefore, the title metal complexes have been screened for their catalytic performances towards ethylene polymerization with the assistance of either methyluminoxane (MAO) or modified methyluminoxane (MMAO).

#### 2.3.1. Ethylene Polymerization by **Co1**–**Co5**

**Co2**/MAO was first selected as a precatalyst system to optimize ethylene polymerization parameters and the results were collected (Table 2). The temperature was the first considered parameter. The polymerization reactivity was significantly increased from 1.89 to 8.32 × 10^6^ g(PE) mol^−1^ (Co) h^−1^ as the temperature elevated from 50 to 70 °C (run 1–3, Table 2), due to the reactant molecule becoming more active at higher temperatures. The polymerization reactivity was then decreased to 4.16 × 10^6^ g(PE) mol^−1^ (Co) h^−1^ (run 4–5, Table 2) with a temperature that continued to increase to 90 °C at a lower concentration of ethylene in toluene and decomposed cobalt active species at this temperature [44].The molecular weight of the polyethylenes obtained reduced from 26.4 to 14.6 kg mol^−1^ as the polymerization temperature increased (run 1–5, Table 2), indicating a faster termination of polymer chains at the increased temperature [45,46].

In comparison to the previous cobalt models described in Scheme 1 (**A**, 60 °C; **B**, 40 °C; **D**, 30 °C), the **Co2** shows a higher thermostability with the optimum temperature (70 °C) due to the positive influence of the bulky substituent at *N*-aryl groups. Moreover, the molecular weights of the resultant polyethylenes are well maintained, with only 7% decreasing within the temperature from 60 to 80 °C.

The parameter of the Al/Co molar ratio was then optimized. The catalytic activity was increased from 1.72 to 8.32 × 10^6^ g(PE) mol^−1^ (Co) h^−1^ as the Al/Co increased from 2000 to 2500, but slightly decreased to 6.98 × 10^6^ g(PE) mol^−1^ (Co) h^−1^ as the ratio further increased to Al/Co = 3000, resulting in the optimized molar ratio of Al/Co = 2500 (run 3, 6–9, Table 2). The molecular weight of the obtained polyethylenes decreased from 24.0 kg mol^−1^ (Al/Co = 2000) to 10.1 kg mol^−1^ (Al/Co = 3000), and the result was similar to previous reports [47,48].

The polymerization was conducted over different running times at 70 °C (run 3, 10–13, Table 2), in order to evaluate the lifetime of the **Co2**/MAO. The catalytic reactivity was quite prominent (13.8 × 10^6^ g(PE) mol^−1^ (Co) h^−1^) at 5 min, suggesting the active species were quickly formed [49]. Then the catalytic activity gradually decreased as the polymerization time prolonged, but still reached up to 4.82 × 10^6^ g(PE) mol^−1^ (Co) h^−1^ at 60 min.

Other precatalysts (**Co1**, **Co3**–**Co5**) were employed to catalyze ethylene polymerization at the optimized polymerization conditions (run 14–17, Table 2). All the complexes showed high activity during ethylene polymerization at 70 °C with an order **Co4** > **Co1** > **Co5** > **Co2** > **Co3**, which was affected by both the electron effect and steric effect [50,51]. **Co4** had most electron-donating methyl groups but the lowest steric hindrance around the metal center resulted in the highest activity while **Co3** with bulky isopropyl groups displayed the lowest activity [30]. **Co5** showed a closed activity with **Co4** and **Co1** for bulky o-sec-phenyl-substituted aryl groups in ligands and donated more electrons [52,53,54]. The molecular weight of the polyethylenes obtained by cobalt complexes was also affected by their ligand structure. The molecular weight of polyethylenes catalyzed by cobalt complexes decreased in the order **Co3** > **Co5** > **Co2** > **Co1** > **Co4**, which was consistent with the literature, which previously reported that the bulky steric substituent of the *N*-aryl group usually produces a higher molecular weight of polyethylenes because bulky substituents prevent the deactivation of active species [46,55]. Moreover, the current model of the cobalt precatalysts displays a higher activity than previous models of cobalt complexes in Scheme 1. For example, the activity of **Co4**/MAO goes up to 10.9 × 10^6^ g(PE) mol^−1^ (Co) h^−1^, while the activity of its analog (**D**) (Scheme 1) is 4.15 × 10^6^ g(PE) mol^−1^ (Co) h^−1^ at their optimized conditions.

**Figure 2:**
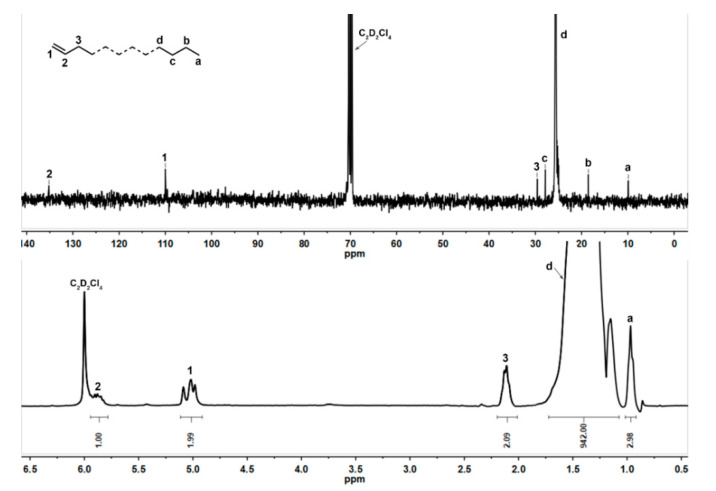
The NMR spectrum of the polyethylenes obtained by **Co2**/MMAO at 70 °C (run 19, Table 2).

The system of the **Co1**–**Co5**/MMAO was employed to further explore the catalytic behavior during the ethylene polymerization process. All of these complexes displayed a high activity at an elevated temperature (70 °C). For the **Co1**–**Co5**/MMAO system, the catalytic activity decreased in the order **Co4** > **Co1** > **Co2** > **Co5** > **Co3**, and the molecular weight of the polyethylenes obtained decreased in the order **Co3** > **Co5** > **Co2** > **Co1** > **Co4**. The melting temperature of all the polyethylenes was around 130 °C, suggesting that polyethylenes had a high linear microstructure [56,57]. To confirm the microstructure of the polyethylenes obtained, the ^1^H NMR and ^13^C NMR of the polyethylenes were carried out and the polyethylenes showed a high linear microstructure with the vinyl-end group (Figure 2). This vinyl-end group was formed by β-H eliminate during ethylene polymerization.

#### 2.3.2. Ethylene Polymerization by **Fe1**–**Fe5**

**Figure 3:**
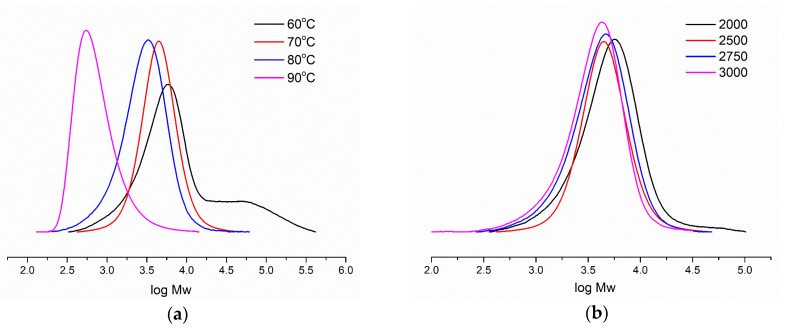
(**a**) GPC curves of the resultant polyethylenes by Fe2/MMAO various temperature (run 1–4, Table 3); (**b**) GPC curves of the resultant polyethylene by Fe2/MMAO various ratio of Al/Fe (run 2 and 5–7, Table 3).

Subsequently, the ethylene polymerizations were conducted by the iron complexes with MMAO as a cocatalyst. Initially, the **Fe2**/MMAO system was used to optimize the polymerization conditions (run 1–4, Table 3). The polymerization activity first increased and then decreased by varying the polymerization temperature from 60 to 90 °C, and the highest activity (16.5 × 10^6^ g(PE) mol^−1^ (Fe) h^−1^) was observed at 70 °C. A better thermostability with an optimized temperature of 70 °C would be a favorable advantage for industrial considerations [57]. From the GPC curves (Figure 3a), the molecular weight decreased as the polymerization temperature increased, and all samples displayed a unimodal distribution of molecular weight except for one sample obtained at 60 °C. The molecular distribution index demonstrated that there was a narrow distribution of molecular weights obtained at a higher temperature (PDI = 1.60–2.45, run 2–4, Table 3) but a broad one observed at a lower temperature (PDI = 4.62, run 1, Table 3). Such a phenomenon was common in catalytic systems—i.e., that there are multi-active species at lower temperatures while single-active species at higher temperatures [58,59]—including bulky substituents.

The polymerization conditions were further optimized by varying the Al/Fe molar ratio from 2000 to 3000 (run 2, 5–7, Table 3). Similar to the previous cobalt complex behavior, the catalytic activity initially increased from 15.8 to 16.5 × 10^6^ g(PE) mol^−1^ (Fe) h^−1^ as the Al/Fe molar ratio increased from 2000 to 2500, and then decreased to 13.0 × 10^6^ g(PE) mol^−1^ (Fe) h^−1^ as the Al/Fe molar ratio further increased to 3000. The molecular weight decreased from 6.64 to 4.31 kg mol^−1^, and all the samples displayed a narrow molecular weight distribution (Figure 3b), highlighting the single active site of the **Fe2**/MMAO system at 70 °C.

The lifetime of **Fe2**/MMAO was assessed by varying the polymerization time from 5 to 60 min (run 6, 8–11, Table 3). It was worth mentioning that a high activity (up to 56.9 × 10^6^ g (PE) mol^−1^ (Fe) h^−1^) was observed at 5 min, indicating that the active species was formed very quickly [57]. By prolonging the polymerization time from 5 to 30 min, the high activity was significantly decreased to 16.5 × 10^6^ g (PE) mol^−1^ (Fe) h^−1^). Additionally, the activity of **Fe2**/MMAO declined to 8.81 × 10^6^ g (PE) mol^−1^ (Fe) h^−1^, further extending the polymerization time to 60 min. Such a result would be attributed to the higher viscosity of the polymerization solution which restricted active species to coordinate with ethylene.

**Figure 4:**
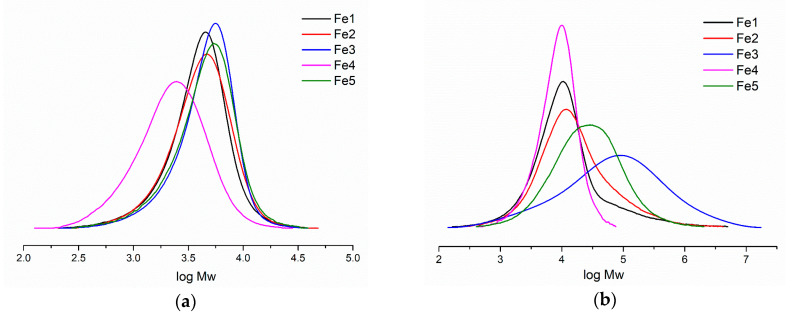
(**a**) GPC curves of the resultant polyethylenes by **Fe1**–**Fe5**/MMAO (run 6, 12-15, Table 3); (**b**) GPC curves of the resultant polyethylene by **Fe1**–**Fe5**/MAO various ratio of Al/Fe (run 16–20, Table 3).

**Fe1**–**Fe5**/MMAO were then employed as catalysts to catalyze ethylene polymerization (run 6, 12–15, Table 3). **Fe1**–**Fe5**/MMAO showed a higher activity (8.81–17.0 × 10^6^ g(PE) mol^−1^ (Fe) h^−1^) at 70 °C, which was higher than most of the previous analogs (**B**–**D**, Scheme 1) (**B**, 70 °C, 9.51 × 10^6^ g(PE) mol^−1^ (Fe) h^−1^; **C**, 70 °C, 10.9 × 10^6^ g(PE) mol^−1^ (Fe) h^−1^; **D**, 70 °C, 15.8 × 10^6^ g(PE) mol^−1^ (Fe) h^−1^; Scheme 1). The activity decreased in the order **Fe4** > **Fe1** ≈ **Fe2** > **Fe5** > **Fe3**, and the order of activity was affected by both the electron effect and steric effect of ligands. All samples showed a really narrow distribution (PDI = 1.48–1.70) with low molecular weights (2.83–5.39 kg mol^−1^, Figure 4a), meaning the single-site active species of **Fe1**–**Fe5**/MMAO. All the polyethylenes show a melting temperature in the range of 129.1–134.6 °C, indicating the highly linear microstructure [56,57]. Their ^1^H NMR and ^13^C NMR spectra at an elevated temperature were measured and demonstrate the linear property of resultant polyethylenes.

**Figure 5:**
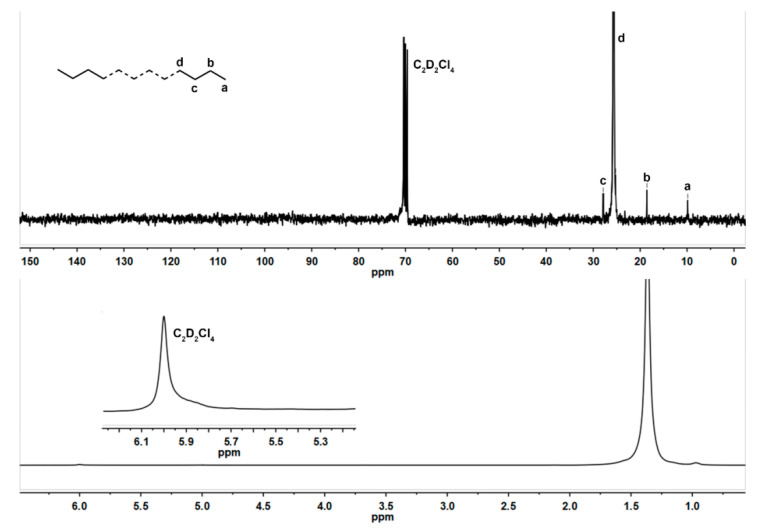
The NMR spectrum of polyethylenes obtained by **Fe2**/MMAO at 70 °C (run 6, Table 3).

The activities of iron precatalysts (8.81–17.0 × 10^6^ g(PE) mol^−1^ (Fe) h^−1^, **Fe1**–**Fe5**/MAO) are generally higher than their cobalt analogs (7.01–10.9 × 10^6^ g(PE) mol^−1^ (Co) h^−1^, **Co1**–**Co5**/MAO). For example, the **Fe2**/MAO at 70 °C reaches 16.7 × 10^6^ g(PE) mol^−1^ (Fe) h^−1^, compared to the **Co2**/MAO with 8.32 × 10^6^ g(PE) mol^−1^ (Co) h^−1^. Meanwhile the resultant polyethylene catalyzed by **Fe2**/MAO has a molecular weight of 4.93 kg mol^−1^, compared with the polyethylene catalyzed by **Co2**/MAO, which has a molecular weight of 19.6 kg mol^−1^. Furthermore, the polyethylene catalyzed by **Co2**/MAO significantly shows a vinyl-end group (Figure 2), but the polyethylene catalyzed by **Fe2**/MAO indicates a saturated end group (Figure 5). The metal center characterizes the microstructure of resultant polyethylene. Herein, polyethylenes with vinyl-ends are favorably produced by cobalt precatalysts.

The **Fe1**–**Fe5**/MAO system was also employed to further investigate the ethylene polymerization behavior (run 16–20, Table 3). The activity decreased in the order **Fe4** > **Fe2** > **Fe5** > **Fe1** > **Fe3** (15.8–2.58 × 10^6^ g(PE) mol^−1^ (Fe) h^−1^), and the molecular weight of polyethylenes decreased in the order **Fe3** > **Fe5** > **Fe2** > **Fe1** > **Fe4** (291–10.7 kg mol^−1^). Different than **Fe1**–**Fe5**/MMAO, the molecular weight of polyethylenes catalyzed by **Fe1**–**Fe5**/MAO was higher with a broader molecular weight distribution. These different ethylene polymerization behaviors affected by cocatalysts may be ascribed to the different active species’ structure nature formed by different cocatalysts [60]. From the GPC curves (Figure 4b), all the samples displayed a unimodal distribution, which illustrates a stable active site during polymerization.

## 3. Materials and Methods

### 3.1. General Considerations

All the experimental manipulations of air- and/or moisture-sensitive compounds were carried out under an atmosphere of nitrogen by the use of standard Schlenk techniques. Freshly distilled toluene was used for the polymerization runs that had previously been dried over sodium for approximately 10 h before distillation under a nitrogen atmosphere. Methylaluminoxane (MAO, 1.46 M in toluene) and modified methylaluminoxane (MMAO, 1.93 M in heptane) were provided by Albemarle Corp (Charlotte, NC, USA). High purity ethylene was provided by the Beijing Yanshan Petrochemical Company and used as received. Other reagents were purchased from Alderich (Beijing, China), Acros (Beijing, China), or local suppliers. The ^1^H and ^13^C NMR spectroscopic measurements for the organic compounds were performed on Bruker DMX 400 MHz instruments (Beijing, China) at room temperature using tetramethylsilane (TMS) as an internal standard. All the chemical shifts and coupling constants are given in ppm and Hz, respectively. Elemental analyses (C, H, and N) were performed on a Flash EA 1112 microanalyzer (Beijing, China). The FT IR spectra were recorded using a PerkinElmer System 2000 FT IR spectrometer (Beijing, China). The molecular weights and molecular weight distributions (*M*_w_/*M*_n_) of the polyethylenes were measured using a PL-GPC220 instrument (Beijing, China) at 150 °C with 1,2,4-trichlorobenzene as the solvent. Data collection and processing were performed using Cirrus GPC Software (Agilent PL-Cirrus Software, Beijing, China) and Multi-Detector Software (Agilent PL-Cirrus Software, Beijing, China). The calibrants employed for construction of the conventional calibration (Polystyrene Calibration KitS-M-10) were provided by PL Company (Beijing, China). The true average molecular weights of the polyethylenes were attained by inputting the Mark–Houwink constants of polyethylenes; K (0.727) and α (40.6) were provided by PL Company (Beijing, China). The sample was dissolved at a concentration of 1.0 mg mL^−1^. The DSC traces and melting points of the polyethylenes were obtained from the second scanning run on a PerkinElmer TA-Q2000 DSC analyzer (Beijing, China) under a nitrogen atmosphere. During the procedure, a sample of about 4.0–6.0 mg was heated to 160 °C at a heating rate of 20 °C min^−1^, followed by 5 min at 150 °C to remove the thermal history and then cooled at a rate of 20 °C min^−1^ to −20 °C. The compound 2-arylimino-6-acetylpyridines (**S1**–**S4**) was prepared according to our previous reports [36,37,38].

### 3.2. Synthesis of 6-Arylimino-2-(2-(1-phenylethyl)naphthalen-1-yl)iminopyridine (**L1**–**L5**)

#### 3.2.1. Synthesis of 6-(2,6-Dimethylphenyl)imino-2-(2-(1-phenylethyl)naphthalen-1-yl)iminopyridine (**L1**)

In this reaction, **S1** (3.4 mmol) and 2-phenethyl-1-naphthylamine (2.9 mmol) were added into a flask with 20 mL of toluene. When the temperature of the reactor reached 110 °C, 0.17 g p-TsOH was added into this reactor. After 6 h, the product was purified by column chromatography on aluminum oxide to afford the product as yellow solid in 14% yield. ^1^H NMR (400 MHz, CDCl_3_, TMS): δ 8.61 (d, *J* = 7.76 Hz, 1H); 8.52 (m, 2H); 7.77 (t, *J* = 7.99, 1H); 7.85 (d, *J* = 8.03 Hz, 1H); 7.59–7.48 (m, 3H); 7.43 (t, *J* = 6.90 Hz, 1H); 7.12–7.08 (m, 8H); 7.0–6.93 (m, 2H); 4.33 (m, 1H), 2.25 (d, *J* = 10.28 Hz, 3H); 2.18 (s, 3H); 2.08 (m, 9H); 1.70 (d, *J* = 7.23 Hz, 3H). ^13^C NMR (100 MHz, CDCl_3_, TMS): δ 170.08, 167.26, 155.25, 148.75, 146.54, 144.86, 136.91, 132.80, 129.30, 128.98, 128.24, 127.91, 127.68, 125.82, 125.62, 125.42, 124.97, 124.03, 123.21, 122.98, 122.64, 122.31, 40.45, 39.08, 21.97, 17.96, 16.45. FT IR (cm^−1^): 3056 (m), 3023 (m), 2962 (m), 2928 (m), 2873 (m), 1698 (s), 1639 (s), 1601 (s), 1565 (s), 1507 (s), 1449 (w), 1361 (s), 1297 (m), 1258 (s), 1233 (m), 1180 (w), 1118 (s), 1094 (m), 1073 (w), 1022 (w), 955 (m), 902 (m), 863 (m), 814 (s), 797 (s), 765 (s), 744 (s), and 697 (s). Anal. Calcd for C_35_H_33_N_3_: C, 84.81, H, 6.71, N, 8.48; found: C, 84.98, H, 6.50, N, 8.52%.

#### 3.2.2. Synthesis of 6-(2,6-Diethylphenyl)imino-2-(2-(1-phenylethyl)naphthalen-1-yl)iminopyridine (**L2**)

Similar to the synthesis **L1**, **L2** was obtained as yellow solid in 18% yield. ^1^H NMR (400 MHz, CDCl_3_, TMS): δ 8.62 (d, *J* = 8.01 Hz, 2H); 8.49 (t, *J* = 7.21 Hz, 1H); 8.20–8.18 (m, 1H); 7.94–7.92 (m, 1H); 7.51–7.41 (m, 4H); 7.26–7.25 (s, 2H); 7.24–7.00 (m, 7H); 4.26 (m, 1H); 2.51–2.49 (m, 4H); 2.16 (d, *J* = 7.22 Hz, 3H); 1.62 (t, *J* = 7.32 Hz, 3H); 1.47 (s, 3H); 1.30–1.06 (m, 6H). ^13^C NMR (100 MHz, CDCl_3_, TMS): δ 170.10, 166.86, 155.26, 147.79, 146.52, 136.92, 132.80, 131.17, 128.98, 128.23, 128.14, 128.04, 127.68, 125.95, 125.81, 125.62, 125.42, 124.98, 124.03, 123.34, 123.20, 122.99, 122.33, 122.27, 40.43, 24.64, 21.95, 16.81, 13.76. FT IR (cm^−1^): 3057 (s), 2964 (s), 2931 (s), 2872 (s), 1632 (s), 1567 (s), 1494 (s), 1453 (w), 1405 (w), 1360 (s), 1321 (m), 1300 (m), 1259 (s), 1246 (s), 1196 (w), 1149 (s), 1122 (s), 1101 (m), 1026 (w), 962 (m), 906 (m), 877 (m), 866 (m), 817 (s), 777 (s), 733 (s), 714 (s). Anal. Calcd for C_37_H_37_N_3_: C, 84.86, H, 7.12, N, 8.02; Found: C, 84.55, H, 7.21, N, 8.24%.

#### 3.2.3. Synthesis of 6-(2,6-Diisopropylphenyl)imino-2-(2-(1-phenylethyl)naphthalen-1-yl)-iminopyridine (**L3**)

Similar to the synthesis of **L1**, **L3** was obtained as a yellow solid in a 15% yield. ^1^H NMR (400 MHz, CDCl_3_, TMS): δ 8.63–8.49 (m, 3H); 8.03–7.95 (m, 2H); 7.69–7.35 (m, 4H); 7.19–7.09 (m, 8H); 4.34 (m, 1H); 2.79 (m, 5H); 1.71 (s, 3H); 1.16 (m, 15H). ^13^C NMR (100 MHz, CDCl_3_, TMS): δ 170.15, 167.08, 155.30, 155.10, 146.54, 144.91, 136.96, 135.83, 132.85, 129.04, 128.27, 128.18, 127.96, 127.72, 125.84, 125.74, 125.66, 125.46, 125.04, 124.08, 123.66, 123.41, 123.26, 123.06, 122.36, 40.45, 39.09, 28.40, 28.38, 28.34, 23.29, 17.16. FT IR (cm^−1^): 3060 (m), 2960 (s), 2925 (m), 2869 (m), 1698 (s), 1632 (s), 1579 (w), 1567 (s), 1497 (s), 1453 (s), 1435 (w), 1361 (s), 1319 (m), 1300 (m), 1238 (s), 1190 (s), 1149 (w), 1121 (s), 1102 (w), 1057 (w), 995 (m), 744 (s), 733 (s), 713 (m), 699 (s), 674 (m). Anal. Calcd for C_39_H_41_N_3_: C, 84.89, H, 7.49, N, 7.62; Found: C, 84.64, H, 7.56, N, 7.80%.

#### 3.2.4. Synthesis of 6-(2,4,6-Trimethylphenyl)imino-2-(2-(1-phenylethyl)naphthalen-1-yl)-iminopyridine (**L4**)

Similar to the synthesis **L1**, **L4** was obtained as yellow solid in an 18% yield. ^1^H NMR (400 MHz, CDCl_3_, TMS): δ 8.64 (d, *J* = 7.12 Hz, 1H); 8.55 (t, *J* = 6.87 Hz, 2H); 8.01 (t, *J* = 7.77 Hz, 1H); 7.88 (d, *J* = 8.08 Hz,1H); 7.69 (m, 2H); 7.59 (t, *J* = 9.09 Hz, 1H); 7.53 (d, *J* = 8.42 Hz, 1H); 7.42 (m, 4H); 7.30 (m, 3H); 4.38 (m, 1H); 2.18 (s, 12H); 1.73 (d, *J* = 7.22 Hz, 3H); 1.61 (s, 3H). ^13^C NMR (100 MHz, CDCl_3_, TMS): δ 170.30, 152.49,145.65. 137.41, 128.72, 128.27, 127.52, 126.38, 125.68, 125.55, 125.01, 124.86, 123.40, 122.69, 120.34, 118.32, 40.18, 25.51, 22.08, 21.77, 18.45, 18.39. FT IR (cm^−1^): 2964 (s), 2900 (s), 1698 (s), 1640 (s), 1565 (s), 1507 (m), 1491 (s), 1449 (s), 1406 (m), 1362 (s), 1297 (m), 1259 (m), 1233 (s), 1074 (m), 1025 (w), 954 (m), 902 (m), 864 (m), 815 (m), 798 (s), 744 (s), 732 (m), 698 (s), 672 (m). Anal. Calcd for C_36_H_35_N_3_: C, 84.83, H, 6.92, N, 8.24; Found: C, 84.77, H, 6.85, N, 8.38%.

#### 3.2.5. Synthesis of 2.6-bis(2-(1-Phenylethyl)naphthalen-1-yl)iminopyridine (**L5**)

The 2,6-diacetylpyridine (1 mmol) and 2-phenethyl-1-naphthylamine (2 mmol) were added into a flask with 20 mL of toluene. When the temperature of the reactor reached 110 °C, 0.17 g of p-TsOH was added into this reactor. After 6 h, the product was purified by column chromatography on aluminum oxide to afford the product as a yellow solid in a 21% yield. ^1^H NMR (400 MHz, CDCl_3_, TMS): 8.09–8.01 (m, 2H); 7.87–7.81 (m, 3H); 7.69–7.35 (m, 10H); 7.16–7.06 (m, 8H); 4.40–4.33 (2H); 1.74–1.72 (s, 6H); 1.54–1.53 (d, *J* = 7.20 Hz, 6H). ^13^C NMR (100 MHz, CDCl_3_, TMS): δ 170.31, 169.62, 146.57, 145.73, 138.74, 137.38, 137.02, 133.11, 132.84, 128.73, 128.47, 128.17, 127.94, 127.71, 127.52, 126.38, 125.80, 125.49, 125.23, 124.94, 124.68, 124.09, 123.39, 122.83, 122.69, 122.55, 120.43, 118.45, 40.61, 40.31, 25.65, 21.78, 16.95. FT IR (cm^−1^): 3026 (m), 2959 (m), 2923 (m), 2870 (m), 1699 (m), 1640 (s), 1566 (m), 1492 (m), 1450 (m), 1413 (m), 1362 (s), 1297 (m), 1235 (s), 1120 (m), 1097 (m), 1067 (m), 1024 (m), 904 (m), 816 (s), 738 (s), 697 (s), 639 (s), 591 (s). Anal. Calcd for C_45_H_39_N_3_: C, 86.92, H, 6.32, N, 6.76; Found: C, 86.98, H, 6.55, N, 6.47%.

### 3.3. Synthesis of 6-Arylimino-2-(2-(1-phenylethyl)naphthalen-1-yl)iminopyridyl-cobalt dichloride (**Co1**–**Co5**)

#### 3.3.1. Synthesis of 6-(2,6-Dimethylphenyl)imino-2-(2-(1-phenylethyl)naphthalen-1-yl)iminopyridine-cobalt dichloride (**Co1**)

In this reaction, **L1** (0.2 mmol), CoCl_2_ 6H_2_O (0.19 mmol) and 10 mL ethanol was added into a Schlenk tube. After a reaction of 6 h, the ethanol was removed and the product was washed by ether. Finally, the product was collected by a filter to afford a yellow solid in a 64% yield. FT IR (cm^−1^): 3399 (s), 3061 (m), 3022 (m), 2967 (m), 2918 (m), 2870 (m), 1685 (w), 1621 (s), 1587 (s), 1508 (m), 1491 (s), 1469 (m), 1450 (m), 1427 (w), 1370 (s), 1308 (w), 1262 (s), 1213 (s), 1162 (w), 1099 (s), 1060 (w), 1026 (w), 904 (s), 817 (s), 768 (s), 746 (s), 700 (s). Anal. Calcd for C_35_H_33_Cl_2_CoN_3_: C, 67.21, H, 5.32, N, 6.72; Found: C, 67.42, H, 5.47, N, 6.64%.

#### 3.3.2. Synthesis of 6-(2,6-Diethylphenyl)imino-2-(2-(1-phenylethyl)naphthalen-1-yl)iminopyridine-cobalt dichloride (**Co2**)

Similar to the synthesis of **Co1**, **Co2** was obtained as yellow solid in 57% yield. FT IR (cm^−1^): 3676 (m), 2969 (s), 2901 (s), 1620 (s), 1584 (s), 1507 (w), 1493 (m), 1449 (s), 1426 (w), 1393 (w), 1372 (s), 1321 (m), 1262 (s), 1208 (s), 1066 (w), 1057 (w), 1026 (s), 976 (w), 904 (m), 868 (s), 808 (s), 768 (s), 745 (s), 702 (s). Anal. Calcd for C_37_H_37_Cl_2_CoN_3_: C, 68.00, H, 5.71, N, 6.43; Found: C, 67.82, H, 5.89, N, 6.53%.

#### 3.3.3. Synthesis of 6-(2,6-Diisopropylphenyl)imino-2-(2-(1-phenylethyl)naphthalen-1-yl)-iminopyridine-cobalt dichloride (**Co3**)

Similar to the synthesis of **Co1**, **Co3** was obtained as a yellow solid in a 68% yield. FT IR (cm^−1^): 3473 (m), 2959 (s), 2922 (m), 2866 (m), 2160 (m), 1619 (s), 1582 (s), 1566 (w), 1508 (s), 1491 (s), 1451 (s), 1371 (s), 1260 (s), 1204 (s), 1176 (m), 1156 (w), 1100 (m), 1058 (m), 1024 (s), 946 (m), 829 (s), 797 (s), 805 (s), 773 (s), 767 (s). Anal. Calcd for C_39_H_41_Cl_2_CoN_3_: C, 68.72, H, 6.06, N, 6.16; Found: C, 68.69, H, 5.94, N, 6.22%.

#### 3.3.4. Synthesis of 6-(2,4,6-Trimethylphenyl)imino-2-(2-(1-phenylethyl)naphthalen-1-yl)iminopyridine-cobalt dichloride (**Co4**)

Similar to the synthesis of **Co1**, **Co4** was obtained as a yellow solid in a 67% yield. FT IR (cm^−1^) 3389 (s), 3061 (w), 3027 (w), 2965 (w), 2918 (w), 1621 (s), 1586 (s), 1567 (w), 1508 (w), 1450 (s), 1428 (w), 1370 (s), 1262 (s), 1220 (s), 1157 (w), 1101 (m), 1061 (m), 1026 (s), 904 (m), 855 (s), 817 (s), 769 (s), 746 (s), 701 (s). Anal. Calcd for C_36_H_35_Cl_2_CoN_3_: C, 67.61, H, 5.52, N, 6.57; Found: C, 67.76, H, 5.74, N, 6.51%.

#### 3.3.5. Synthesis of 2.6-bis((2-(1-phenylethyl)naphthalen-1-yl)iminopyridine-cobalt dichloride (**Co5**)

Similar to the synthesis of **Co1**, **Co5** was obtained as a yellow solid in a 68% yield. FT IR (cm^−1^): 3473 (m), 3065 (m), 2959 (s), 2922 (m), 2865 (m), 2160 (m), 1619 (s), 1582 (s), 1567 (w), 1508 (m), 1491 (w), 1451 (s), 1430 (w), 1372 (s), 1336 (w), 1321 (w), 1311 (w), 1260 (s), 1205 (s), 1176 (w), 1156 (w), 1100 (m), 1058 (m), 1025 (s), 976 (w), 946 (w), 905 (m), 866 (m), 829 (s), 814 (s), 805 (s), 797 (s), 767 (s), 747 (s), 714 (s), 704 (s). Anal. Calcd for C_45_H_39_Cl_2_CoN_3_: C, 71.91, H, 5.23, N, 5.59; Found: C, 69.98, H, 5.24, N, 5.47%.

### 3.4. Synthesis of 6-Arylimino-2-(2-(1-phenylethyl)naphthalen-1-yl)iminopyridyl-iron dichloride (**Fe1**–**Fe5**)

#### 3.4.1. Synthesis of 6-(2,6-Dimethylphenyl)imino-2-(2-(1-phenylethyl)naphthalen-1-yl)iminopyridine-iron dichloride (**Fe1**)

In this reaction, **L1** (0.2 mmol), FeCl_2_4H_2_O (0.19 mmol) and 10 mL of ethanol was added into a Schlenk tube. After a reaction of 6 h, the ethanol was removed and the product was washed by ether. Finally, the product was collected by a filter to afford blue solid in 41% yield. FT IR (cm^−1^): 3420 (s), 3066 (m), 2968 (m), 2932 (m), 2157 (w), 1647 (w), 1621 (s), 1589 (s), 1491 (s), 1450 (s), 1370 (s), 1337 (w), 1263 (m), 1203 (s), 1108 (w), 1060 (m), 1026 (m), 910 (w), 769 (s), 748 (s), 714 (s), 701 (s). Anal. Calcd for C_35_H_33_Cl_2_FeN_3_: C, 67.54, H, 5.34, N, 6.75; Found: C, 67.39, H, 5.74, N, 6.51%.

#### 3.4.2. Synthesis of 6-(2,6-Diethylphenyl)imino-2-(2-(1-phenylethyl)naphthalen-1-yl)iminopyridine-iron dichloride (**Fe2**)

Similar to the synthesis of **Fe1**, **Fe2** was obtained as a blue solid in a 75% yield. FT IR (cm^−1^): 2970 (s), 2900 (s), 1621 (s), 1587 (s), 1584 (s), 1491 (s), 1449 (s), 1370 (s), 1262 (m), 1208 (s), 1057 (m), 1027 (m), 905 (w), 807 (m), 823 (m), 768 (s), 749 (s). Anal. Calcd for C_37_H_37_Cl_2_FeN_3_: C, 68.32, H, 5.73, N, 6.46; Found: C, 68.39, H, 5.86, N, 6.67%.

#### 3.4.3. Synthesis of 6-(2,6-Diisopropylphenyl)imino-2-(2-(1-phenylethyl)naphthalen-1-yl)-iminopyridine-iron dichloride (**Fe3**)

Similar to the synthesis of **Fe1**, **Fe3** was obtained as a blue solid in a 90% yield. FT IR (cm^−1^): 2962 (s), 2900 (s), 1615 (s), 1580 (s), 1491 (s), 1450 (s), 1423 (m), 1372 (s), 1308 (m), 1268 (m), 1203 (s), 1099 (m), 1058 (m), 1027 (m), 828 (m), 812 (m), 797 (w), 767 (s), 748 (s), 714 (m), 704 (s). Anal. Calcd. for C_39_H_41_Cl_2_FeN_3_: C, 69.04, H, 6.09, N, 6.19; Found: C, 69.18, H, 5.97, N, 6.25%.

#### 3.4.4. Synthesis of 6-(2,4,6-Trimethylphenyl)imino-2-(2-(1-phenylethyl)naphthalen-1-yl)-iminopyridine-iron dichloride (**Fe4**)

Similar to the synthesis of **Fe1**, **Fe4** was obtained as a blue solid in a 46% yield. FT IR (cm^−1^): 2971 (s), 2901 (s), 1620 (s), 1588 (s), 1508 (m), 1492 (s), 1407 (m), 1394 (w), 1370 (s), 1250 (m), 1204 (s), 1066 (m), 1028 (m), 903 (m), 822 (m), 748 (s), 701 (s). Anal. Calcd. for C_36_H_35_Cl_2_FeN_3_: C, 67.94, H, 5.54, N, 6.60; Found: C, 67.87, H, 5.65, N, 6.72%.

#### 3.4.5. Synthesis of 2.6-bis(2-(1-phenylethyl)naphthalen-1-yl)iminopridine-iron dichloride (**Fe5**)

Similar to the synthesis of **Fe1**, **Fe5** was obtained as a blue solid in a 55% yield. FT IR (cm^−1^): 3058 (m), 3025 (m), 2960 (m), 2922 (m), 2868 (m), 1838 (s), 1619 (s), 1574 (s), 1492 (s), 1430 (s), 1365 (m), 1299 (s), 1241 (m), 1207 (w), 1132 (w), 1108 (m), 1077 (m), 1030 (m), 998 (m), 964 (w), 909 (w), 872 (m), 813 (w), 781 (s), 741 (s), 697 (s). Anal. Calcd for C_45_H_39_Cl_2_FeN_3_: C, 72.20, H, 5.25, N, 5.61; Found: C, 72.34, H, 5.48, N, 5.63%.

### 3.5. X-ray Crystallographic Studies

Single crystals of **Co3** and **Co5** suitable for the X-ray diffraction analysis were obtained by layering a dichloromethane solution of the corresponding complex with ethyl ether at room temperature under a nitrogen atmosphere. With graphite monochromated Mo-Kα radiation (λ = 0.71073 Å) at 170.00(10)K or CuKα (λ = 1.54184 Å) at 169.99(14)K, the cell parameters were obtained by the global refinement of the positions of all collected reflections. The intensities were corrected for Lorentz and polarization effects and empirical absorption. The structures were solved by direct methods and refined by full-matrix least-squares on *F^2^*. All hydrogen atoms were placed in calculated positions. Structure solution and structure refinement were performed using the SHELXT-97 package [61,62]. The free solvent molecules present within the crystal structures were removed by using the SQUEEZE option of the crystallographic program PLATON [61,62]. Detail of the X-ray structure determinations and refinements are provided in Table 4. X-ray crystallographic data in Calibration Index File (CIF) for the Cambridge Crystallographic Data Centre (CCDC) 2024895 (**Co2**) and 2024896 (**Co5**) are available free of charge from the Cambridge Crystallographic Data Centre.

### 3.6. General Procedure for Ethylene Polymerization under 10 Atm Pressure

The polymerization at an ethylene pressure of 10 atm was carried out in a 250 mL stainless steel autoclave (Dalian Sanling Electronic Manufacture, Dalian, China) equipped with an ethylene pressure control system, a mechanical stirrer, and a temperature controller. The autoclave was evacuated and backfilled with ethylene three times. When the required temperature was reached, the precatalyst (2.0 µmol) was dissolved in toluene (25 mL) in a Schlenk tube and injected into the autoclave containing ethylene (1 atm) followed by the addition of more toluene (25 mL). The required amount of cocatalyst (MAO and MMAO) and additional toluene was 100 mL. The autoclave was immediately pressurized with an ethylene pressure of 10 atm and the stirring commenced. After the required reaction time, the reactor was cooled with a water bath and the ethylene pressure vented. Following quenching of the reaction with 10% hydrochloric acid in ethanol, the polymer was collected and washed with ethanol and dried with a vacuum oven at 60 °C and weighed.

## 4. Conclusions

A series of iron and cobalt complexes supporting by 6-arylimino-2-(2-(1-phenylethyl) naphthalen-1-yl)iminopyridine ligands (**L1**–**L5**) were synthesized and characterized. On the activation with either MMAO or MAO, cobalt precatalysts showed high activities up to 10.9 × 10^6^ g(PE) mol^−1^ (Co) h^−1^ at an elevated temperature. Their activities are in the order as **Co4** > **Co1** > **Co5** > **Co2** > **Co3**, meanwhile the molecular weights of the resultant polyethylenes decreased in the order **Co3** > **Co5** > **Co2** > **Co1** > **Co4**, which was interpreted as the electron-donating ligands enhancing the catalytic activity and the steric hindrance decreasing the catalytic activity, producing polyethylene with a higher molecular weight. In comparison to the cobalt precatalysts, their iron analogs **Fe1**–**Fe5**/MMAO displayed higher activities up to 17.0 × 10^6^ g(PE) mol^−1^ (Fe) h^−1^ when polymerizing ethylene and produced highly linear polyethylenes with lower molecular weights in the range of kg mol^−1^ and with a narrow polydispersity. In the case of using MAO as the cocatalyst, the polyethylenes obtained by **Fe1**–**Fe5**/MAO showed higher molecular weights up to 291 kg mol^−1^ with boarder polydispersity, indicating the multispecies of active sites. All precatalysts achieved a good thermostability and were positively influenced by the new modifications of the tridentate ligands.

## Figures and Tables

**Table 1:** Selected bond lengths and angles for **Co3** and **Co5**.

	Co3	Co5		Co3	Co5
Bond Lengths (Å)					
Co1-N1	2.170(4)	2.294(4)	Co1-Cl1	2.3093(16)	2.2511(15)
Co1-N2	2.050(4)	2.021(4)	Co1-Cl2	2.2555(16)	2.2357(14)
CO1-N3	2.196(4)	2.212(4)			
Bond Angles (deg)					
N1-Co1-N2	74.16(16)	73.56(15)	N2-Co1-Cl1	89.35(12)	120.48(13)
N1-Co1-N3	141.24(15)	149.89(14)	N2-Co1-Cl2	152.82(13)	124.83(13)
N1-Co1-Cl1	101.50(12)	96.36(11)	N3-Co1-Cl1	99.67(12)	98.87(12)
N1-Co1-Cl2	100.67(12)	96.71(11)	N3-Co1-Cl2	97.61(12)	100.34(11)
N2-Co1-N3	74.01(17)	76.32(16)	Cl1-Co1-Cl2	117.74(7)	114.48(6)

**Table 2:** Polymerization screening using **Co1**–**Co5** ^a^.

Run	Precat.	Al/Co	T (°C)	t (min)	PE (g)	Act. ^b^	*M*_w_^c^	*M*_w_/*M*_n_^c^	*T*_m_ (°C) ^d^
1	**Co2**	2500	50	30	1.89	1.89	26.4	4.86	131.8
2	**Co2**	2500	60	30	4.23	4.23	20.2	4.77	130.4
3	**Co2**	2500	70	30	8.32	8.32	19.6	4.41	131.4
4	**Co2**	2500	80	30	5.74	5.74	18.8	4.83	130.8
5	**Co2**	2500	90	30	4.16	4.16	14.6	4.53	131.6
6	**Co2**	2000	70	30	1.72	1.72	24.0	4.82	130.0
7	**Co2**	2250	70	30	4.81	4.81	21.1	4.96	131.8
8	**Co2**	2750	70	30	7.15	7.15	19.4	4.11	131.4
9	**Co2**	3000	70	30	6.98	6.98	10.1	3.34	131.3
10	**Co2**	2500	70	5	3.44	13.8	18.8	4.68	131.1
11	**Co2**	2500	70	15	5.19	10.4	18.9	4.46	131.0
12	**Co2**	2500	70	45	9.23	6.16	21.1	4.62	131.7
13	**Co2**	2500	70	60	9.64	4.82	23.8	4.75	130.7
14	**Co1**	2500	70	30	10.2	10.2	15.1	6.56	129.3
15	**Co3**	2500	70	30	7.01	7.01	22.6	2.19	131.8
16	**Co4**	2500	70	30	10.9	10.9	14.3	6.50	128.9
17	**Co5**	2500	70	30	9.24	9.24	21.8	4.90	131.0
18 ^e^	**Co1**	2500	70	30	5.27	5.27	17.1	6.39	130.2
19 ^e^	**Co2**	2500	70	30	4.71	4.71	21.8	4.26	130.9
20 ^e^	**Co3**	2500	70	30	2.91	2.91	25.6	2.63	133.4
21 ^e^	**Co4**	2500	70	30	5.61	5.61	17.0	6.27	129.9
22 ^e^	**Co5**	2500	70	30	4.68	4.68	23.4	4.82	131.8

^a^ Conditions: 2 µmol cobalt, 100 mL toluene, 10 atm C_2_H_4_, methyluminoxane (MAO) as cocatal. ^b^ Values in units of 10^6^ g(PE) mol^−1^ (Co) h^−1^. ^c^ Determined using GPC, *M*_w_: kg mol^−1^. ^d^ Determined using DSC; ^e^ methyluminoxane (MMAO) as cocatal.

**Table 3:** Polymerization screening using **Fe1**–**Fe5** ^a^.

Run	Precat.	Al/Fe	T (°C)	t (min)	PE (g)	Act. ^b^	*M*_w_^c^	*M*_w_/*M*_n_^c^	*T*_m_ (°C) ^d^
1	**Fe2**	2500	60	30	16.4	16.4	20.8	4.62	131.7
2	**Fe2**	2500	70	30	16.5	16.5	9.01	2.45	130.4
3	**Fe2**	2500	80	30	9.74	9.74	3.59	1.60	130.1
4	**Fe2**	2500	90	30	0.630	0.630	1.08	1.68	128.2
5	**Fe2**	2000	70	30	15.8	15.8	6.64	1.73	130.5
6	**Fe2**	2750	70	30	16.7	16.7	4.93	1.55	130.0
7	**Fe2**	3000	70	30	13.0	13.0	4.31	1.56	129.0
8	**Fe2**	2750	70	5	9.48	56.9	4.24	1.55	128.0
9	**Fe2**	2750	70	15	12.8	25.6	4.54	1.60	129.2
10	**Fe2**	2750	70	45	17.5	11.6	4.84	1.57	129.8
11	**Fe2**	2750	70	60	17.6	8.81	5.78	1.50	130.5
12	**Fe1**	2750	70	30	16.5	16.5	4.72	1.54	129.1
13	**Fe3**	2750	70	30	8.81	8.81	5.32	1.48	129.8
14	**Fe4**	2750	70	30	17.0	17.0	2.83	1.70	129.2
15	**Fe5**	2750	70	30	15.5	15.5	5.39	1.56	129.4
16 ^e^	**Fe1**	2750	70	30	9.05	9.05	43.2	7.16	132.6
17 ^e^	**Fe2**	2750	70	30	14.4	14.4	49.0	6.69	132.8
18 ^e^	**Fe3**	2750	70	30	2.58	2.58	291	27.8	134.6
19 ^e^	**Fe4**	2750	70	30	15.8	15.8	10.7	1.69	131.0
20 ^e^	**Fe5**	2750	70	30	9.29	9.29	56.7	4.71	132.5

^a^ Conditions: 2 µmol iron complexes, 100 mL toluene, 10 atm C_2_H_4_, MAO as cocatal; ^b^ Values in units of 10^6^ g(PE) mol^−1^ (Fe) h^−1^. ^c^ Determined using GPC, *M*_w_: kg mol^−1^. ^d^ Determined using DSC; ^e^ MMAO as cocatal.

**Table 4:** Crystal data and structure refinement for **Co3** and **Co5**.

	Co3	Co5
CCDC No.	2024895	2024896
Empirical formula	C_78_H_82_Cl_4_CoN_6_	C_45_H_39_Cl_2_CoN_3_
Formula weight	1363.15	751.62
Temperature/K	169.99(14)	170.00(10)
Crystal system	triclinic	monoclinic
Space group	P1	P2_1_/c
a/Å	10.9022(2)	13.0704(7)
b/Å	11.4855(2)	19.0887(9)
c/Å	17.2387(3)	16.5809(7)
α/°	74.805(2)	90
β/°	74.746(2)	98.492(4)
γ/°	68.494(2)	90
Volume (Å^3^)	1904.26(7)	4091.5(3)
Z	1	4
D_Calcd._ (g cm^−3^)	1.189	1.220
μ (mm ^−1^)	5.034	0.583
*F*(000)	714.0	1564.0
Crystal size (mm^3^)	0.5 × 0.19 × 0.15	0.3 × 0.25 × 0.19
Radiation	CuKα (λ = 1.54184)	MoKα (λ = 0.71073)
2Θ range (°)	5.406 to 151.256	6.868 to 57.49
Index ranges	−13 ≤ h ≤ 13, −14 ≤ k ≤ 14, −21 ≤ l ≤ 16	−16≤ h ≤15, −22 ≤ k ≤ 25, −22 ≤ l ≤ 18
Reflections collected	23545	34131
Independent reflections	10720 [R_int_ = 0.0381, R_sigma_ = 0.0431]	9259 [R_int_ = 0.0740, R_sigma_ = 0.0817]
Data/restraints/parameters	10720/3/825	9259/0/464
Goodness of fit on *F*^2^	1.040	1.031
Final R indexes [I ≥ 2σ (I)]	R_1_ = 0.0454, wR_2_ = 0.1119	R_1_ = 0.0852, wR_2_ = 0.2379
Final R indexes (all data)	R_1_ = 0.0505, wR_2_ = 0.1217	R_1_ = 0.1302, wR_2_ = 0.2606
Largest diff. peak/hole (e Å^−3^)	0.59/−0.35	1.39/−0.68

## Supplementary Materials

The following are available online at https://www.mdpi.com/1420-3049/25/18/4244/s1, X-ray crystallographic data for **Co3** and **Co5**. CCDC: 2024895 (**Co3**), 2024896 (**Co5)**.

## Abbreviations

ORTEP	Oak Ridge Thermal Ellipsoid Plot
CIF	Calibration Index File
GPC	Gel Permeation Chromatography
MAO	Methylauminoxane
MMAO	Modified methylaluminoxane
PDI	Polydispersity index
*T*m	Melting temperature

## References

[1] Small B.L., Brookhart M., Bennett A.M.A. (1998). Highly active iron and cobalt catalysts for the polymerization of ethylene. J. Am. Chem. Soc..

[2] Britovsek G.J.P., Gibson V.C., Kimberley B.S., Maddox P.J., McTavish S.J., Solan G.A., White A.J.P., Williams D.J. (1998). Novel olefin polymerization catalysts based on iron and cobalt. Chem. Commun..

[3] Britovsek G.J.P., Bruce M., Gibson V.C., Kimberley B.S., Maddox P.J., Mastroianni S., McTavish S.J., Redshaw C., Solan G.A., Stromberg S. (1999). Iron and cobalt ethylene polymerization catalysts bearing 2,6-bis(imino)pyridyl ligands: Synthesis, structures, and polymerization studies. J. Am. Chem. Soc..

[4] Gibson V.C., Redshaw C., Solan G.A. (2007). Bis(imino)pyridines:  Surprisingly Reactive Ligands and a Gateway to New Families of Catalysts. Chem. Rev..

[5] Zhang L., Hou X., Yu J., Chen X., Hao X., Sun W.-H. (2011). 2-(R-1H-Benzoimidazol-2-yl)-6-(1-aryliminoethyl) pyridyliron(II) dichlorides: Synthesis, characterization and the ethylene oligomerization behavior. Inorg. Chim. Acta.

[6] Suo H., Solan G.A., Ma Y., Sun W.-H. (2018). Developments in compartmentalized bimetallic transition metal ethylene polymerization catalysts. Coord. Chem. Rev..

[7] Johnson L.K., Killian C.M., Brookhart M. (1995). New Pd(II)- and Ni(II)- Based Catalysts for Polymerization of Ethylene and α-Olefins. J. Am. Chem. Soc..

[8] Killian C.M., Tempel D.J., Johnson L.K., Brookhart M. (1996). Living Polymerization of α-Olefins Using Ni(II) α-Diimine Catalysts. Synthesis of New Block Polymers Based on α-Olefins. J. Am. Chem. Soc..

[9] Vignesh A., Zhang Q., Ma Y., Liang T., Sun W.-H. (2020). Attaining highly branched polyethylene elastomers by employing modified α-diiminonickel(II) catalysts: Probing the effects of enhancing fluorine atom on the ligand framework towards mechanical properties of polyethylene. Polymer.

[10] Yuan S.-F., Fan Z., Zhang Q., Flisak Z., Ma Y., Sun Y., Sun W.-H. (2020). Enhancing performance of α-diiminonickel precatalyst for ethylene polymerization by substitution with the 2,4-bis(4,4′-dimethoxybenzhydryl)-6-methylphenyl group. Appl. Organomet. Chem..

[11] Zada M., Vignesh A., Guo L., Zhang R., Zhang W., Ma Y., Sun Y., Sun W.-H. (2020). Sterically and Electronically Modified Aryliminopyridyl-Nickel Bromide Precatalysts for an Access to Branched Polyethylene with Vinyl/Vinylene End Groups. ACS Omega.

[12] Suo H., Chen Q., Zhang W., Ma Y., Sun W.-H. (2020). Methylene-bridged bis(8-arylimino)-5,6,7-trihydro- quinolylnickel precatalysts for ethylene polymerization. J. Polym. Sci..

[13] Yue E., Zeng Y., Zhang W., Sun Y., Cao X.-P., Sun W.-H. (2016). Highly linear polyethylenes using the 2-(1-(2,4-dibenzhydrylnaphthylimino)ethyl)-6-(1-(arylimino)ethyl)-pyridylcobalt chlorides: Synthesis, characterization and ethylene polymerization. Sci. China Chem..

[14] Guo L., Zada M., Zhang W., Vignesh A., Zhu D., Ma Y., Liang T., Sun W.-H. (2019). Highly linear polyethylenes tailored with 2,6-bis 1-(p-dibenzo-cycloheptylarylimino)ethyl pyridylcobalt dichlorides. Dalton Trans..

[15] Han M., Zhang Q., Oleynik I.I., Suo H., Solan G.A., Oleynik I.V., Ma Y., Liang T., Sun W.-H. (2020). High molecular weight polyethylenes of narrow dispersity promoted using bis(arylimino)cyclohepta[b] pyridine-cobalt catalysts ortho-substituted with benzhydryl & cycloalkyl groups. Dalton Trans..

[16] Zada M., Vignesh A., Suo H., Ma Y., Liu H., Sun W.-H. (2020). NNN-type iron (II) complexes consisting sterically hindered dibenzocycloheptyl group: Synthesis and catalytic activity towards ethylene polymerization. Mol. Catal..

[17] Zhang Q., Wu N., Xiang J., Solan G.A., Suo H., Ma Y., Liang T., Sun W.-H. (2020). Bis-cycloheptyl-fused bis(imino)pyridine-cobalt catalysts for PE wax formation: Positive effects of fluoride substitution on catalytic performance and thermal stability. Dalton Trans..

[18] Suo H., Li Z., Oleynik I.V., Wang Z., Oleynik I.I., Ma Y., Liu Q., Sun W.-H. (2020). Achieving strictly linear polyethylenes by the NNN-Fe precatalysts finely tuned with different sizes of ortho-cycloalkyl substituents. Appl. Organomet. Chem..

[19] Ivanchev S.S., Yakimanskii A.V., Rogozin D.G. (2003). Quantum-chemical calculation of the thermal stability of bis(imine) and bis(imino)pyridine catalysts of ethylene polymerization. Quantum Chem. Calc..

[20] Semikolenova N.V., Zakharov V.A., Echevskaja L.G., Matsko M.A., Bryliakov K.P., Talsi E.P. (2009). Homogeneous catalysts for ethylene polymerization based on bis(imino)pyridine complexes of iron, cobalt, vanadium and chromium. Catal. Today.

[21] Chen Y., Qian C., Sun J. (2003). Fluoro-Substituted 2,6-Bis(imino)pyridyl Iron and Cobalt Complexes:  High-Activity Ethylene Oligomerization Catalysts. Organometallics.

[22] Xie G., Li T., Zhang A. (2010). Highly active and selective ethylene oligomerization catalysts: Asymmetric 2,6-bis(imino)pyridyl iron (II) complexes with alkyl and halogen substitutients. Inorg. Chem. Commun..

[23] Zhao W., Yu J., Song S., Yang W., Liu H., Hao X., Redshaw C., Sun W.-H. (2012). Controlling the ethylene polymerization parameters in iron pre-catalysts of the type 2-1-(2,4-dibenzhydryl-6-methylphenylimino) ethyl-6-1-(arylimino)ethyl pyridyliron dichloride. Polymer.

[24] Wang S., Li B., Liang T., Redshaw C., Li Y., Sun W.-H. (2013). Synthesis, characterization and catalytic behavior toward ethylene of 2-1-(4,6-dimethyl-2-benzhydryl-phenylimino)ethyl-6-1-(arylimino)ethyl -pyridylmetal (iron or cobalt) chlorides. Dalton Trans..

[25] Chen Y., Yang W., Sha R., Fu R.-D., Sun W.-H. (2014). Correlating net charges and the activity of bis(imino)pyridylcobalt complexes in ethylene polymerization. Inorg. Chim. Acta.

[26] Zhao W., Yue E., Wang X., Yang W., Chen Y., Hao X., Cao X., Sun W.-H. (2017). Activity and stability spontaneously enhanced toward ethylene polymerization by employing 2-(1-(2,4-dibenzhydrylnaphthyl imino)ethyl)-6-(1-(Arylimino)ethyl)pyridyliron(II) dichlorides. J. Polym. Sci. A Polym. Chem..

[27] Ma Z., Yang W., Sun W.-H. (2017). Recent Progress on Transition Metal (Fe, Co, Ni, Ti and V) Complex Catalysts in Olefin Polymerization with High Thermal Stability. Chin. J. Chem..

[28] Chen Q., Zhang W., Solan G.A., Zhang R., Guo L., Hao X., Sun W.-H. (2018). CH(phenol)-Bridged Bis(imino)pyridines as Compartmental Supports for Diiron Precatalysts for Ethylene Polymerization: Exploring Cooperative Effects on Performance. Organometallics.

[29] Zhang Q., Ma Y., Suo H., Solan G.A., Liang T., Sun W.-H. (2019). Co-catalyst effects on the thermal stability/activity of N,N,N-Co ethylene polymerization catalysts bearing fluoro-substituted N-2,6-dibenzhydrylphenyl groups. Appl. Organomet. Chem..

[30] Mitchell N.E., Anderson W.C., Long J.B.K. (2017). Mitigating chain-transfer and enhancing the thermal stability of co-based olefin polymerization catalysts through sterically demanding ligands. J. Polym. Sci. A Polym. Chem..

[31] Zhang R., Han M., Ma Y., Solan G.A., Liang T., Sun W.-H. (2019). Steric and electronic modulation of iron catalysts as a route to remarkably high molecular weight linear polyethylenes. Dalton Trans..

[32] Yu J., Liu H., Zhang W., Hao X., Sun W.-H. (2011). Access to highly active and thermally stable iron procatalysts using bulky 2-1-(2,6-dibenzhydryl-4-methylphenylimino)ethyl-6-1-(arylimino)ethyl pyridine ligands. Chem. Commun..

[33] Yu J., Huang W., Wang L., Redshaw C., Sun W.-H. (2011). 2-1-(2,6-Dibenzhydryl-4-methylphenylimino)ethyl -6-1-(arylimino)ethylpyridylcobalt(II) dichlorides: Synthesis, characterization and ethylene polymerization behavior. Dalton Trans..

[34] Ivanchev S.S., Yakimansky A.V., Rogozin D.G. (2004). Quantum-chemical calculations of the effect of cycloaliphatic groups in alpha-diimine and bis(imino)pyridine ethylene polymerization precatalysts on their stabilities with respect to deactivation reactions. Polymer.

[35] Lin W., Zhang L., Suo H., Vignesh A., Yousuf N., Hao X., Sun W.-H. (2020). Synthesis of characteristic polyisoprenes using rationally designed iminopyridyl metal (Fe and Co) precatalysts: Investigation of co-catalysts and steric influence on their catalytic activity. New J. Chem..

[36] Ma Z., Sun W.-H., Li Z.-L., Shao C.-X., Hu Y.-L., Li X.-H. (2002). Ethylene polymerization by iron complexes with symmetrical and unsymmetrical ligands. Polym. Int..

[37] Kaul F.A.R., Puchta G.T., Frey G.D., Herdtweck E., Herrmann W.A. (2007). Iminopyridine Complexes of 3d Metals for Ethylene Polymerization:  Comparative Structural Studies and Ligand Size Controlled Chain Termination. Organometallics.

[38] Sun W.-H., Zhao W., Yu J., Zhang W., Hao X., Redshaw C. (2012). Enhancing the activity and thermal stability of iron precatalysts using 2-(1-{2,6-bis[bis(4-fluorophenyl)methyl]-4-methylphenylimino}ethyl)- 6-[1-(arylimino)ethyl]pyridines. Macromol. Chem. Phys..

[39] Mahrnood Q., Guo J., Zhang W., Ma Y., Liang T., Sun W.-H. (2018). Concurrently improving the thermal stability and activity of ferrous precatalysts for the production of saturated/unsaturated polyethylene. Organometallics.

[40] Zhuang R., Li H., Wang H., Liu H., Dong B., Zhao W., Hu Y., Zhang X. (2018). Cobalt (II) complexes bearing N,N,S tridentate ligands as precatalysts for 1,3-butadiene polymerization. Inorg. Chim. Acta.

[41] Zhang R., Huang Y., Solan G.A., Zhang W., Hu X., Hao X., Sun W.-H. (2019). Gem-Dimethyl-substituted bis(imino)dihydroquinolines as thermally stable supports for highly active cobalt catalysts that produce linear PE waxes. Dalton Trans..

[42] Khoshsefat M., Dechal A., Ahmadjo S., Mortazavi S.M.M., Zohuri G., Soares J.B.P. (2019). Amorphous to high crystalline PE made by mono and dinuclear Fe-based catalysts. Eur. Polym. J..

[43] Bariashir C., Wang Z., Ma Y., Vignesh A., Hao X., Sun W.-H. (2019). Finely tuned α,α′-bis(arylimino)-2,3:5,6-bis(pentamethylene)pyridine-based practical iron precatalysts for targeting highly linear and narrow dispersive polyethylene waxes with vinyl ends. Organometallics.

[44] Chandran D., Kwak C.H., Oh J.M., Ahn I.Y., Ha C.-S., Kim I. (2008). Ethylene oligomerizations by sterically modulated salicylaldimine cobalt(II) complexes combined with various alkyl aluminum cocatalysts. Catal. Lett..

[45] Wang Z., Ma Y., Guo J., Liu Q., Solan G.A., Liang T., Sun W.-H. (2019). Bis(imino) pyridines fused with 6-and 7-membered carbocylic rings as N,N,N-scaffolds for cobalt ethylene polymerization catalysts. Dalton Trans..

[46] Wang Z., Zhang R., Zhang W., Solan G.A., Liu Q., Liang T., Sun W.-H. (2019). Enhancing thermostability of iron ethylene polymerization catalysts through N,N,N-chelation of doubly fused bis(arylimino)-2,3:5,6- bis(hexamethylene)pyridines. Catal. Sci. Technol..

[47] Du S., Wang X., Zhang W., Flisak Z., Sun Y., Sun W.-H. (2016). A practical ethylene polymerization for vinyl-polyethylenes: Synthesis, characterization and catalytic behavior of α,α′-bisimino-2,3:5,6-bis (pentamethylene)pyridyliron chlorides. Polym. Chem..

[48] Mahmood Q., Yue E., Guo J., Zhang W., Ma Y., Hao X., Sun W.-H. (2018). Nitro-functionalized bis(imino)pyridylferrous chlorides as thermo-stable precatalysts for linear polyethylenes with high molecular weights. Polymer.

[49] Huang C., Zhang Y., Solan G.A., Ma Y., Hu X., Sun Y., Sun W.-H. (2017). Vinyl-Polyethylene Waxes with Narrow Dispersity Obtained by Using a Thermally Robust Bis(imino)trihydroquinolyl chromium Catalyst. Eur. J. Inorg. Chem..

[50] Yi J., Yang W., Sun W.-H. (2016). Quantitative Investigation of the Electronic and Steric Influences on Ethylene Oligo/Polymerization by 2-Azacyclyl-6-aryliminopyridylmetal (Fe, Co, and Cr) Complexes. Macromol. Chem. Phys..

[51] Yang W., Ma Z., Sun W.-H. (2016). Modeling study on the catalytic activities of 2-imino-1,10-phenanthrolinylmetal (Fe, Co, and Ni) precatalysts in ethylene oligomerization. RSC Adv..

[52] Abu-Surrah A.S., Lappalainen K., Piironen U., Lehmus P., Repo T., Leskelä M. (2002). New bis(imino)pyridine-iron(II)- and cobalt(II)-based catalysts: Synthesis, characterization and activity towards polymerization of ethylene. J. Organomet. Chem..

[53] Cao X., He F., Zhao W., Cai Z., Hao X., Shiono T., Redshaw C., Sun W.-H. (2012). 2-[1-(2,6-Dibenzhydryl-4-chlorophenylimino)ethyl]-6-[1-(arylimino)ethyl]pyridyliron(II) dichlorides: Synthesis, characterization and ethylene polymerization behavior. Polymer.

[54] Huang C., Du S., Solan G.A., Sun Y., Sun W.-H. (2017). From polyethylene waxes to HDPE using an α,α′-bis(arylimino)-2,3:5,6-bis(pentamethylene)pyridyl-chromium(iii) chloride pre-catalyst in ethylene polymerisation. Dalton Trans..

[55] Akcakaviran D., Mauder D., Hess C., Sievers T.K., Kurth D.G., Shenderovich I., Limbach H.-H., Findenegg G.H. (2008). Carboxylic Acid-Doped SBA-15 Silica as a Host for Metallo-supramolecular Coordination Polymers. J. Phys. Chem. B.

[56] Zhang W., Chai W., Sun W.-H., Hu X., Redshaw C., Hao X. (2012). 2-(1-(Arylimino)ethyl)-8-arylimino- 5,6,7-trihydroquinoline iron(II) chloride complexes: Synthesis, characterization, and ethylene polymerization behavior. Organometallics.

[57] Zhang W., Wang S., Du S., Guo C.-Y., Hao X., Sun W.-H. (2014). 2-(1-(2,4-Bis((di(4-fluorophenyl)methyl)-6- methylphenylimino)ethyl)-6-(1-(arylimino)ethyl)pyridylmetal (iron or cobalt) complexes: Synthesis, characterization, and ethylene polymerization behavior. Macromol. Chem. Phys..

[58] Semikolenova N.V., Sun W.-H., Soshnikov I.E., Matsko M.A., Kolesova O.V., Zakharov V.A., Bryliakov K.P. (2017). Origin of "multisite-like" ethylene polymerization behavior of the single-site nonsymmetrical bis(imino)pyridine iron(II) complex in the presence of modified methylaluminoxane. ACS Catal..

[59] Wang Z., Solan G.A., Mahmood Q., Liu Q., Ma Y., Hao X., Sun W.-H. (2018). Bis(imino)pyridines incorporating doubly fused eight-membered rings as conformationally flexible supports for cobalt ethylene polymerization catalysts. Organometallics.

[60] Bryliakov K.P., Talsi E.P., Semikolenova N.V., Zakharov V.A. (2009). Formation and nature of the active sites in bis(imino)pyridine iron-based polymerization catalysts. Organometallics.

[61] Sheldrick G.M. (2015). SHELXT-integrated space-group and crystal-structure determination. Acta Crystallogr. Sect. A Struct. Chem..

[62] Sheldrick G.M. (2015). Crystal structure refinement with SHELXL. Acta Crystallogr. Sect. C Struct. Chem..

