# Supplementary material for: 6-Arylimino-2-(2-(1-phenylethyl)naphthalen-1-yl)-iminopyridylmetal (Fe and Co) Complexes as Highly Active Precatalysts for Ethylene Polymerization: Influence of Metal and/or Substituents on the Active, Thermostable Performance of Their Complexes and Resultant Polyethylenes
Source: Molecules. 2020 Sep 16;25(18):4244. doi: 10.3390/molecules25184244 (PMC7570845; doi:10.3390/molecules25184244)

checkCIF/PLATON report

You have not supplied any structure factors. As a result the full set of tests cannot be run.

THIS REPORT IS FOR GUIDANCE ONLY. IF USED AS PART OF A REVIEW PROCEDURE FOR PUBLICATION, IT SHOULD NOT REPLACE THE EXPERTISE OF AN EXPERIENCED CRYSTALLOGRAPHIC REFEREE.

No syntax errors found. CIF dictionary Interpreting this report

Datablock: Co3

```
Bond precision:  C-C = 0.0099 A          Wavelength=1.54184

Cell:          a=10.9022(2)      b=11.4855(2)     c=17.2387(3)
               alpha=74.805(2)   beta=74.746(2)   gamma=68.494(2)
Temperature:   170 K
```

	Calculated	Reported
Volume	1904.26(7)	1904.26(7)
Space group	P 1	P 1
Hall group	P 1	P 1
Moiety formula	C39 H41 Cl2 Co N3 [+ solvent]	2(C39 H41 Cl2 Co N3)
Sum formula	C39 H41 Cl2 Co N3 [+ solvent]	C78 H82 Cl4 Co2 N6
Mr	681.58	1363.15
Dx,g cm-3	1.189	1.189
Z	2	1
Mu (mm-1)	5.034	5.034
F000	714.0	714.0
F000’	712.29	
h,k,lmax	13,14,21	13,14,21
Nref	15836[7918]	10720
Tmin,Tmax	0.365,0.470	0.320,1.000
Tmin’	0.070	

```
Correction method= # Reported T Limits: Tmin=0.320 Tmax=1.000
AbsCorr = MULTI-SCAN

Data completeness= 1.35/0.68      Theta(max)= 75.628

R(reflections)= 0.0454( 10102)    wR2(reflections)= 0.1217( 10720)

S = 1.040                  Npar= 825
```

```
The following ALERTS were generated. Each ALERT has the format
       test-name_ALERT_alert-type_alert-level.
Click on the hyperlinks for more details of the test.
```

Alert level C

```
PLAT220_ALERT_2_C NonSolvent   Resd 1  C   Ueq(max)/Ueq(min) Range        3.5 Ratio
PLAT234_ALERT_4_C Large Hirshfeld Difference C25      --C26      .       0.16 Ang.
PLAT341_ALERT_3_C Low Bond Precision on  C-C Bonds ...............    0.00991 Ang.
```

Alert level G

```
PLAT012_ALERT_1_G N.O.K.   _shelx_res_checksum Found in CIF ......     Please Check
PLAT013_ALERT_1_G N.O.K.   _shelx_hkl_checksum Found in CIF ......     Please Check
PLAT014_ALERT_1_G N.O.K.   _shelx_fab_checksum Found in CIF ......     Please Check
PLAT042_ALERT_1_G Calc. and Reported MoietyFormula Strings  Differ     Please Check
PLAT045_ALERT_1_G Calculated and Reported Z Differ by a Factor ...       2.00 Check
PLAT154_ALERT_1_G The s.u.'s on the Cell Angles are Equal ..(Note)      0.002 Degree
PLAT606_ALERT_4_G Solvent Accessible VOID(S) in Structure ........          ! Info
PLAT791_ALERT_4_G Model has Chirality at C20         (Sohnke SpGr)          S Verify
PLAT791_ALERT_4_G Model has Chirality at C59         (Sohnke SpGr)          R Verify
PLAT794_ALERT_5_G Tentative Bond Valency for Co1       (II)      .       2.00 Info
PLAT794_ALERT_5_G Tentative Bond Valency for Co2       (II)      .       2.04 Info
PLAT850_ALERT_4_G Check Flack Parameter Exact Value 0.00 with s.u.       0.00 Check
PLAT869_ALERT_4_G ALERTS Related to the Use of SQUEEZE Suppressed           ! Info
PLAT933_ALERT_2_G Number of OMIT Records in Embedded .res File ...          3 Note
PLAT941_ALERT_3_G Average HKL Measurement Multiplicity ...........        3.1 Low
```

```
   0 ALERT level A = Most likely a serious problem - resolve or explain
   0 ALERT level B = A potentially serious problem, consider carefully
   3 ALERT level C = Check. Ensure it is not caused by an omission or oversight
  15 ALERT level G = General information/check it is not something unexpected

   6 ALERT type 1 CIF construction/syntax error, inconsistent or missing data
   2 ALERT type 2 Indicator that the structure model may be wrong or deficient
   2 ALERT type 3 Indicator that the structure quality may be low
   6 ALERT type 4 Improvement, methodology, query or suggestion
   2 ALERT type 5 Informative message, check
```

Datablock: Co5

```
Bond precision:  C-C = 0.0088 A          Wavelength=0.71073

Cell:           a=13.0704(7)     b=19.0887(9)       c=16.5809(7)
                alpha=90         beta=98.492(4)     gamma=90
Temperature:    170 K
```

	Calculated	Reported
Volume	4091.5(3)	4091.5(3)
Space group	P 21/c	P 1 21/c 1
Hall group	-P 2ybc	-P 2ybc
Moiety formula	C45 H39 Cl2 Co N3 [+ solvent]	C45 H39 Cl2 Co N3
Sum formula	C45 H39 Cl2 Co N3 [+ solvent]	C45 H39 Cl2 Co N3
Mr	751.62	751.62
Dx,g cm-3	1.220	1.220
Z	4	4
Mu (mm-1)	0.583	0.583
F000	1564.0	1564.0
F000’	1567.01	
h,k,lmax	17,25,22	16,25,22
Nref	10623	9259
Tmin,Tmax	0.840,0.895	0.495,1.000
Tmin’	0.840	

Correction method= # Reported T Limits: Tmin=0.495 Tmax=1.000
AbsCorr = MULTI-SCAN

Data completeness= 0.872 Theta(max)= 28.745

R(reflections)= 0.0852(5832) wR2(reflections)= 0.2606(9259)

S = 1.031 Npar= 464

The following ALERTS were generated. Each ALERT has the format
test-name_ALERT_alert-type_alert-level.
Click on the hyperlinks for more details of the test.

Alert level C

PLAT084_ALERT_3_C High wR2 Value (i.e. > 0.25) .... 0.26 Report
PLAT094_ALERT_2_C Ratio of Maximum / Minimum Residual Density .... 2.02 Report
PLAT241_ALERT_2_C High 'MainMol' Ueq as Compared to Neighbors of C24 Check
PLAT341_ALERT_3_C Low Bond Precision on C-C Bonds .... 0.00881 Ang.

Alert level G

PLAT012_ALERT_1_G N.O.K. _shelx_res_checksum Found in CIF ...... Please Check
PLAT013_ALERT_1_G N.O.K. _shelx_hkl_checksum Found in CIF ...... Please Check
PLAT014_ALERT_1_G N.O.K. _shelx_fab_checksum Found in CIF ...... Please Check
PLAT072_ALERT_2_G SHELXL First Parameter in WGHT Unusually Large 0.13 Report
PLAT083_ALERT_2_G SHELXL Second Parameter in WGHT Unusually Large 6.64 Why ?
PLAT605_ALERT_4_G Largest Solvent Accessible VOID in the Structure 113 A**3
PLAT793_ALERT_4_G Model has Chirality at C20 (Centro SPGR) R Verify
PLAT793_ALERT_4_G Model has Chirality at C38 (Centro SPGR) R Verify
PLAT794_ALERT_5_G Tentative Bond Valency for Co1 (II) . 2.05 Info
PLAT869_ALERT_4_G ALERTS Related to the Use of SQUEEZE Suppressed ! Info
PLAT941_ALERT_3_G Average HKL Measurement Multiplicity .... 3.7 Low

```
 0 ALERT level A = Most likely a serious problem - resolve or explain
 0 ALERT level B = A potentially serious problem, consider carefully
 4 ALERT level C = Check. Ensure it is not caused by an omission or oversight
11 ALERT level G = General information/check it is not something unexpected

 3 ALERT type 1 CIF construction/syntax error, inconsistent or missing data
 4 ALERT type 2 Indicator that the structure model may be wrong or deficient
 3 ALERT type 3 Indicator that the structure quality may be low
 4 ALERT type 4 Improvement, methodology, query or suggestion
 1 ALERT type 5 Informative message, check
```

It is advisable to attempt to resolve as many as possible of the alerts in all categories. Often the minor alerts point to easily fixed oversights, errors and omissions in your CIF or refinement strategy, so attention to these fine details can be worthwhile. In order to resolve some of the more serious problems it may be necessary to carry out additional measurements or structure refinements. However, the purpose of your study may justify the reported deviations and the more serious of these should normally be commented upon in the discussion or experimental section of a paper or in the "special_details" fields of the CIF. checkCIF was carefully designed to identify outliers and unusual parameters, but every test has its limitations and alerts that are not important in a particular case may appear. Conversely, the absence of alerts does not guarantee there are no aspects of the results needing attention. It is up to the individual to critically assess their own results and, if necessary, seek expert advice.

Publication of your CIF in IUCr journals

A basic structural check has been run on your CIF. These basic checks will be run on all CIFs submitted for publication in IUCr journals (*Acta Crystallographica*, *Journal of Applied Crystallography*, *Journal of Synchrotron Radiation*); however, if you intend to submit to *Acta Crystallographica Section C* or *E* or *IUCrData*, you should make sure that full publication checks are run on the final version of your CIF prior to submission.

Publication of your CIF in other journals

Please refer to the *Notes for Authors* of the relevant journal for any special instructions relating to CIF submission.

PLATON version of 10/08/2020; check.def file version of 06/08/2020

Datablock Co3 - ellipsoid plot

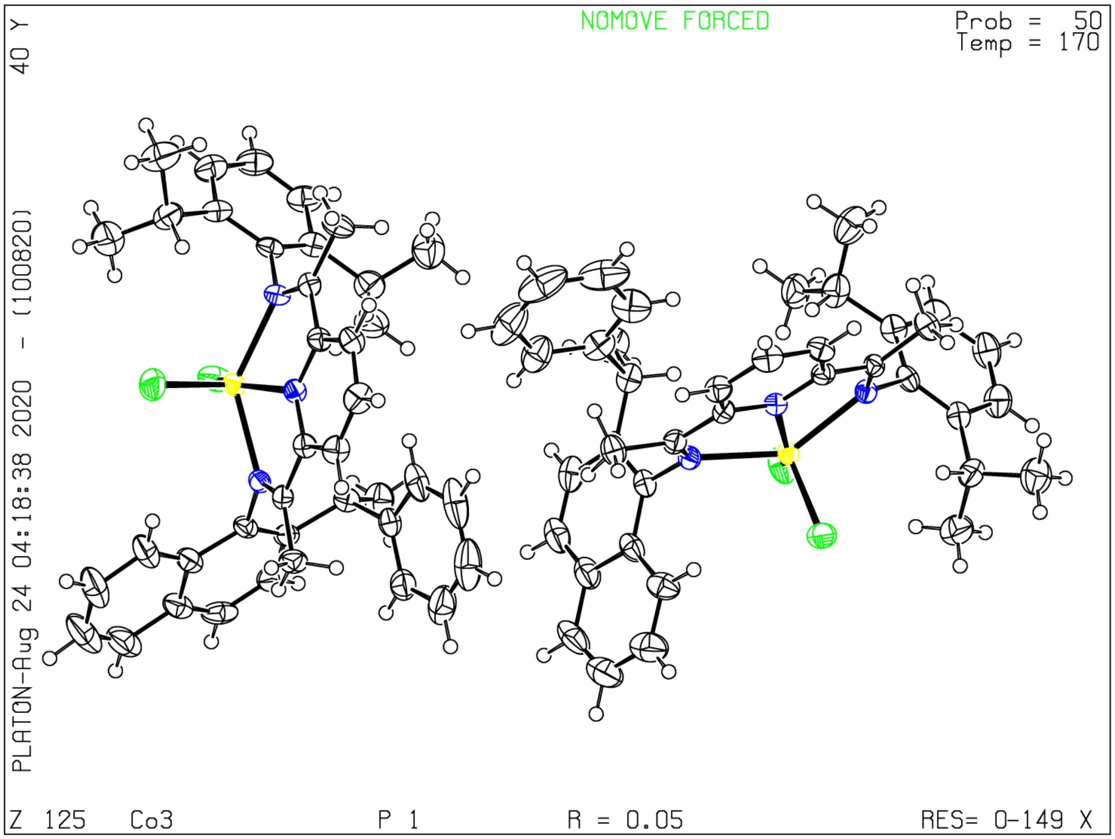

Datablock Co5 - ellipsoid plot

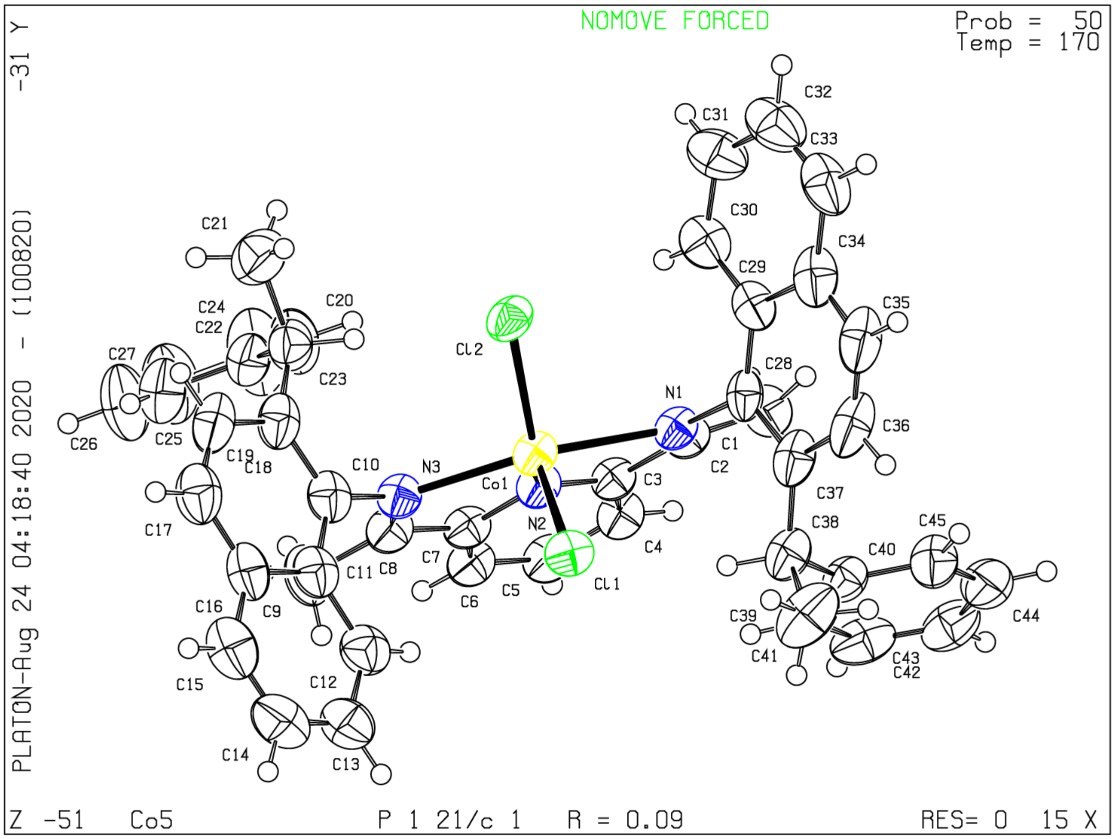

Supplement: Supplementary file 1 [file molecules-25-04244-s001.pdf]
